# Proximate Composition, Antioxidant Properties, and Hepatoprotective Activity of Three Species of Shellfish of the Pacific Coast of Russia

**DOI:** 10.3390/molecules26113397

**Published:** 2021-06-03

**Authors:** Ekaterina P. Karaulova, Evgeny V. Yakush, Tatiana N. Slutskaya, Lidiya V. Shulgina

**Affiliations:** Russian Federal Research Institute of Fisheries and Oceanography, Pacific Branch (TINRO), 4, Shevchenko Alley, 690091 Vladivostok, Russia; evyakush@mail.ru (E.V.Y.); t.slutskaya@mail.ru (T.N.S.); lvshulgina@mail.ru (L.V.S.)

**Keywords:** Pacific region, shellfish, radical scavenging activity, hepatoprotective activity

## Abstract

The objective of the present study was to investigate the proximate composition, antiradical properties and hepatoprotective activity of three species of shellfish, *Corbicula japonica*, *Spisula sachalinensis*, and *Anadara broughtonii*, from the coastal areas of Far East Russia. Biologically active peptides such as taurine (3.74 g/100 g protein) and ornithine (2.12 g/100 g protein) have been found in the tissues of *A. broughtonii*. *C. japonica* contains a high amount of ornithine (5.57 g/100 g protein) and taurine (0.85 g/100 g protein). The maximum DPPH and ABTS radical scavenging activity (36.0 µg ascorbic acid/g protein and 0.68 µmol/Trolox equiv/g protein, respectively) was determined for the tissue of *C. japonica*. The protein and peptide molecular weight distribution of the shellfish tissue water extracts was investigated using HPLC. It was found that the amount of low molecular weight proteins and peptides were significantly and positively correlated with radical scavenging activity (Pearson’s correlation coefficient = 0.96), while the amount of high molecular weight proteins negatively correlated with radical scavenging activity (Pearson’s correlation coefficient = −0.86). Hepatoprotective activity, measured by the survival rate of HepG2 hepatocytes after cotreatment with t-BHP, was detected for *C. japonica*. The highest protection (95.3 ± 2.4%) was achieved by the cold water extract of *C. japonica* at the concentration of 200 mg/mL. Moreover, oral administration of hot water extract of *C. japonica* to rats before the treatment with CCl_4_ exhibited a markedly protective effect by lowering serum levels of ALT and AST, inhibiting the changes in biochemical parameters of functional state of rat liver, including MDA, SOD, GSH and GST.

## 1. Introduction

Currently, there is significant consumption of natural resources, resulting in irreversible change and the collapse of traditional food stocks [1,2]. Therefore, it is necessary to search for new potential sources of food in biologically active substances and consider their use, while maintaining the ecological balance.

Marine organisms are a source of natural food products, new drugs, and biologically active compounds, covering a wide range of substances of different origins [3].

The marine environment possibly have molecules and compounds with different structures and vastly different substances from the natural products in terrestrial organisms [4]. Extreme conditions lead organisms to evolve, creating bioactive compounds for such purposes as reproduction, communication, and protection against predation, infection, and competition [5] in order to survive in the environment. Therefore, there is a huge possibility to encounter a whole new set of natural compounds from marine organisms [6].

Shellfish represent a conspicuous component of marine ecosystems. Bivalves in the coastal waters of the Sea of Japan are a source of proteins, amino acids, and biologically active components. The technological and biochemical features of clams differ significantly from traditional raw fish materials [7,8,9].

Marine bivalve mollusks can serve as a source of functional materials and bioactive peptides. These peptides can be used as functional food ingredients or as nutraceuticals and pharmaceuticals to improve human health and prevent disease. There are several reports of the presence of molecules with health promoting potential in different marine bivalves [4,8,10].

More than 200 marine bivalve species inhabit the Far Eastern seas. In the northern part of the Sea of Japan, there are from 120 to 130 species; and in its southern part, up to about 200 species. Some of them, such as *Patinopecten yessoesis* and *Crenomytilus grayanus*, are extensively used in the food industry. Others, such as *Anadara broughtonii*, *Corbicula japonica*, and *Spisula sachalinensis* are potentially suitable for food use.

The clams of the genus Corbicula are a group of small freshwater mollusks that are distributed worldwide. *C. japonica*, belonging to the family Corbiculidae, is distributed in the brackish and freshwater estuaries and lagoons. These clams form large aggregations in salt lakes and the brackish water bays of the Primorsky, Khabarovsk, and Sakhalin regions. Due to their nutritional value, these mollusks are important fishing species [11].

The bivalve mollusk *A. broughtonii* is an Asian Pacific upper subtidal species. The northern border of its geographical range passes across Peter the Great Bay., The ark shell is the most marketable object of commercial fishing among the bivalve mollusks of Primorye. Active coastal fishing for this species in the Russian Far East has been conducted since 1996 [12].

The Sakhalin surf clam (*S. sachalinensis*) is one of the dominant species among the upper sublittoral mollusks in the Primorsky region. This species is distributed in the northern part of the Sea of Japan from the shores of Korea and Possiet Bay to the Olga Bay. This is a commercial species [13].

Unfortunately, these species of shellfish are not traditional food for the population in Russia and are used as food in the Far East of Russia only. However, these species are commercial and caught for sale in Japan, Korea, and China [14,15]. Currently, shellfish is considered as a most important source of protein and bioactive peptides [4]. These proteins and peptides may be used as functional ingredients or simply as nutritional additives to low protein quality food. New integrative research of the biological activity of bivalve tissues will be the impetus for the introduction of functional nutrition forms of marine products into the human diet.

In this study, shows original data on the biochemical composition and nutraceutical properties of three bivalve species (*A. broughtoni*, *C. japonica*, and *S. sachalinensis*) from the Russian part of the Sea of Japan. The qualitative and quantitative composition, antioxidant activity, and analysis of hepatoprotective activity were investigated to assess the biochemical values of these bivalves as putative food with functional properties. The present study was undertaken to evaluate, for the first time, the antioxidant potentials and hepatoprotective activity of three of the most exported Russian bivalve species, *A. broughtoni, C. japonica,* and *S. sachalinensis*.

## 2. Results

### 2.1. Proximate Composition of Muscle Tissue of Bivalve Mollusks

The results of the proximate composition determined for the bivalve mollusks muscle tissue are shown in Table 1. *C. japonica* demonstrated a high moisture content (89.0%) and the lowest protein content of all three studied species (38.1%). *S. sachalinensis* and *A. broughtonii* had protein contents that were comparable or slightly lower than the values found in several different commercial shellfish species. *S. sachalinensis* and *A. broughtonii* had satisfactory protein/lipid ratios (approximately 15:1), which is of particular interest from a nutritional point of view, since proteins are valuable nitrogen and amino acid sources for the human body. *C. japonica* had a higher crude fat level (33.2%) compared with the other clam tissues, including commercial species (*Patinopecten yessoesis* and *Crenomytilus grayanus*). *A. broughtonii* had carbohydrate level (6.1%), which is lower than those reported for several clam species, resulting in a low energetic value. For comparison, the carbohydrate content of the tissues of commercial shellfish (*Patinopecten yessoesis* and *Crenomytilus grayanus*) is 16–18%. The ash level was approximately the same for all clam species (8.6–10.1%). The tissues of the bivalve caught after spawning (September−October) contain 3 to 4 times less protein, 4 to 5 times less carbohydrates and 10 to 12% more moisture.

### 2.2. Amino Acid Composition

The amino acid (AA) profile of the bivalve mollusks is summarized in Table 2. Generally, *C. japonica*, *A. broughtonii*, and *S. sachalinensis* demonstrated similar amino acid profiles. Other than histidine, phenylalanine, and methionine contents, we found no significant differences between the species. Leucine and lysine were the most abundant amino acids in all samples with relatively high contents of isoleucine, threonine, and valine, while the contents of tyrosine and histidine were very low. The nutritional quality of a protein source can be evaluated from its essential amino acid score (AAS). The AAS compares the levels of essential amino acids (EAAs) in the clam tissue with FAO/WHO (2007) [16] recommended protein standards.

The AAS indicated that all amino acids (other than methionine) were present in adequate or excess quantities. An elevated ratio of hydrophobic amino acids (isoleucine, methionine, phenylalanine, and valine) reportedly improves antioxidant activities [17], as well as cytotoxic and anticancer effects [18]. Thus, these bivalve mollusks are a good source of EAAs for the production of functional foods with desirable biological activities. All the EAAs of these proteins meet with FAO/WHO suggested requirements.

Unique free amino acids were found in bivalve tissue. So, in addition to essential amino acids, the muscle tissues of *A. broughtonii* contained such AAs as glutamic acid (22.46 g/100 g protein), histidine (2.15 g/100 g protein), alanine (6.37 g/100 g protein), and glycine (13.54 g/100 g protein). Biologically active substances such as taurine (3.74 g/100 g protein) and ornithine (2.12 g/100 g protein) have been found in the tissues of *A. broughtonii*. *C. japonica* contains high amounts of ornithine (5.57 g/100 g protein) and taurine (0.85 g/100 g protein).

### 2.3. Mineral Content

The concentrations of Ca, Mg, Na, K, Fe, Mn, Zn, and Cu on a wet weight basis are shown in Table 3. All the mineral concentrations determined in this work were at reasonable levels. All shellfish samples investigated in the work were shown to be good sources of K, Ca, Fe, and Zn. The contents of K, Ca, Mg, and Na in *C. japonica* were significantly higher than those in *A. broughtonii* and *S. sachalinensis* (*p* < 0.05). This indicates that *C. japonica* represents a complementary source of these minerals. The content of the trace metal iron was highest in *A. broughtonii*, at 65.2 µg/g (*p* < 0.05). The zinc content was highest in *C. japonica* at 45.6 µg/g and lowest in the *A. broughtonii* and *S. sachalinensis* at 12.4 µg/g and 13.2 µg/g, respectively (*p* < 0.05).

### 2.4. 2,2-Diphenyl-1-Picrylhydrazyl Radical Scavenging Activity (DPPH) of Water Extracts of Bivalve Tissue

**Figure 1:**
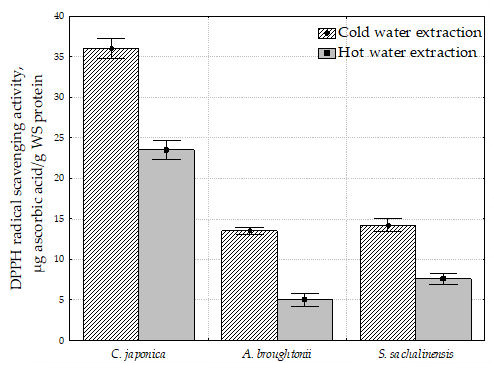
DPPH radical scavenging activity of water-soluble proteins of bivalve tissue.

Since extraction is usually the first step involved in the analysis of water animal bioactive compounds, this study investigated the feasibility of cold and hot water extraction for the recovery of proteins from bivalve tissues. The bivalve tissue were extracted by cold and hot water at temperatures of 4 ± 2 °C and 95 ± 3 °C, respectively. The DPPH radical scavenging activity of water extracts of bivalve tissue is shown in Figure 1. The DPPH radical scavenging activity was expressed in µg ascorbic acid/g water-soluble (WS) protein.

A negative correlation between the DPPH radical scavenging activity and the total amount of tissue proteins was found. The highest radical scavenging activity (36.0 µg ascorbic acid/g WS protein) of *C. japonica* tissue had a lower proportion of the total amount of protein (38.1%). On the other hand, the *A. broughtonii* tissue showed a low radical scavenging effect (13.5 µg ascorbic acid/g WS protein) with the highest proportion of the total amount of protein (81.9%). The radical scavenging activity of *S. sachalinensis* was characterized as 14.2 µg ascorbic acid/g WS protein with a total protein amount of 61.1%. At the same time, the amount of water-soluble proteins in all mollusks studied was approximately the same and varied from 20.3 for *C. japonica* to 21.3 for *A. broughtonii*.

When the extraction was performed at high temperature, the DPPH radical scavenging activity decreased by 34, 62, and 46% for *C. japonica*, *A. broughtonii*, and *S. sachalinensis*, respectively. However, the content of water-soluble proteins increased slightly. Apparently, the total amount of WS proteins and peptides of bivalve tissue does not affect their DPPH radical scavenging activity.

### 2.5. 2,2′-Azinobis(3-Ethylbenzothiazoline-6-Sulfonic Acid) Diammonium Salt Radical Scavenging Activity (ABTS) of Water Extracts of Bivalve Tissue

**Figure 2:**
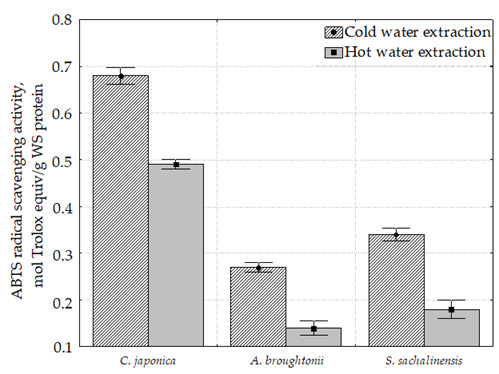
ABTS radical scavenging activity of water-soluble proteins of bivalve tissue.

All samples were characterized by the presence of ABTS radical scavenging activity (Figure 2). A positive correlation was found between radical scavenging activity measured with DPPH and ABTS; Pearson’s correlation coefficient was 0.99, the relationship was statistically significant (*p* < 0.005).

As it can be seen, the highest ABTS radical scavenging activity was determined in the *C. japonica* cold and hot water extract (0.68 and 0.49 µmol Trolox equiv/g, respectively). Hot water extracts of *C. japonica*, *A. broughtonii* and *S. sachalinensis* were characterized by the decreased ABTS radical scavenging activity compared to the cold extracts.

The maximum difference between the cold water and hot water extracts for radical scavenging activity was observed in *A. broughtonii* (2.7 times for DPPH and 1.9 times for ABTS). The minimum difference between the hot water and cold water extracts for radical scavenging activity was observed in *C. japonica* (1.5 times for DPPH and 1.4 times for ABTS).

### 2.6. Molecular Weight Distribution of Water-Soluble Proteins and Peptides of Bivalve Tissue

**Figure 3:**
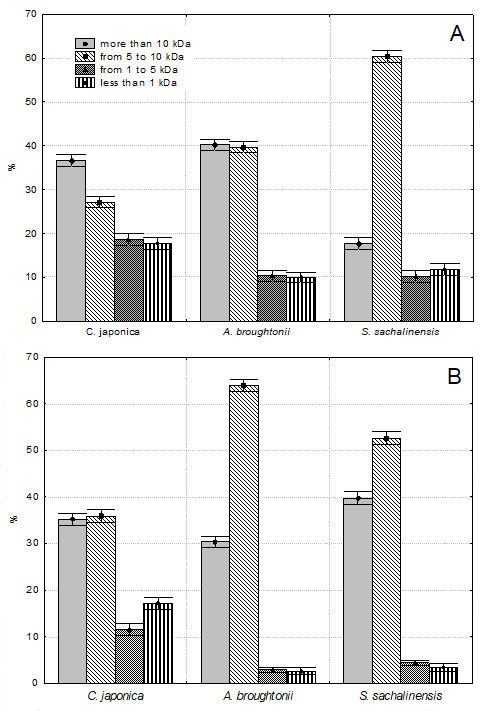
Molecular weight distribution of water-soluble proteins and peptides of cold (**A**) and hot (**B**) water extract of bivalve tissue.

The molecular weight distribution of water-soluble proteins of water extracts was estimated using high performance liquid chromatography (HPLC). Based on the results of gel filtration chromatography on TSKgel G 3000PWXL column, we divided the peaks on the chromatograms of water extracts into four groups: more than 10 kDa; from 5 to 10 kDa; from 1 to 5 kDa and less than 1 kDa. Figure 3A,B demonstrates the area percentages of these peak groups in the gel filtration chromatograms of cold and hot water extracts of bivalves. HPLC results and molecular weight distribution of cold and hot water extracts of bivalves are shown in the Supplementary Materials (Figures S1–S6, Tables S1–S6).

As shown from the analysis of Figure 3, the peptide and protein size distribution was different. It was observed that in all the samples, the total area percentage of Groups I and II was the largest, from 63.7 (*C. japonica*) to 94.3% (*A. broughtonii)*. The total area percentage of Groups III and VI was much smaller, from 5.7 (*A. broughtonii)* to 36.3% (*C. japonica*).

A significant decrease in low molecular weight proteins during hot water extraction was detected. The proportion of protein and peptide components of Group III and IV were lower in hot water extracts by 20.7% for *C. japonica*, by 64.4% for *S. sachalinensis* and by 71.8% for *A. broughtonii*. The total area percentage of low molecular weight peptides (with a molecular weight of less than 5 kDa) was the largest for *C. japonica* and amounted to 36.3 and 28.8% for cold and hot extracts, respectively. It should be noted that peptides with molecular weight from 1 to 5 kDa were present only in water extracts of *C. japonica*. Peptides with molecular weight from 2 to 4 kDa were found in water extracts of *A. broughtonii* and *S. sachalinensis*.

### 2.7. Relationship between the Protein Composition and the Radical Scavenging Activity of Water Extract of Bivalve Tissue

In order to understand the effect of the protein composition of water extracts on their antioxidant activities, we correlated the four peak groups in the gel filtration chromatograms of the water extracts of bivalve tissue to the antioxidant properties. The results are shown in Table 4.

It was found that the amount of Group III and Group IV was significantly and positively correlated with the radical scavenging activity, while the amount of Group II negatively correlated with the radical scavenging activity. This data revealed that low molecular weight protein components corresponded to a higher level of antiradical activity. In the present study, the *C. japonica* water extracts revealed the most potent DPPH and ABTS scavenging ability, compared with other bivalve extracts. The *C. japonica* cold water extract was characterized by the highest amount of low molecular weight peptides, and its antiradical activity against DPPH and ATBS was the highest.

To confirm the conclusion about the correlation, we fractionated substances with different molecular weights (Groups I, II, III and IV) using the fraction collector. The amount of DPPH radical scavenging activity for each group is shown in Table 5. It was shown that fractions of substances with a molecular weight of more than 10 kDa did not have radical scavenging activity. The activity of Group II was maximal and amounted to 50.0–65.8% of the total activity of the extracts.

### 2.8. In Vitro Hepatoprotective Activity of Water Extracts of Bivalve Tissue

**Figure 4:**
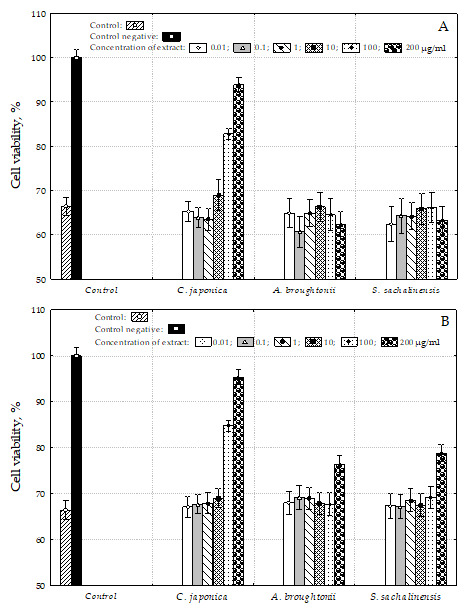
Effect of water bivalve extracts on cytotoxicity of t-BHP-induced HepG2 cells. (**A**) Hot water extracts; (**B**) cold water extracts; control—control sample, untreated; control negative-treated with t-BHP (0.4 mM).

To confirm the radical scavenging activity as well as the hypothesis of hepatoprotective activity of water bivalve extracts, HepG2 cells were co-treated with tert-butyl hydroperoxide (t-BHP) and different concentrations of water bivalve extracts. As shown in Figure 4A,B, the viability of HepG2 cells treated by 0.4 mM t-BHP alone decreased to 66.4% of the control group. Same bivalve water extracts prevented t-BHP-induced cell death (*p* < 0.05), and the cytotoxicity-inhibitory activity was dependent on the concentration of the sample.

As shown in Figure 4, *C. japonica* cold and hot water extract treatment over 100 μg/mL concentration significantly increased the cell viability in t-BHP-treated HepG2 cells in a dose-dependent manner (*p* < 0.05). The cell viabilities were shown to be comparable to the control level in treatment with a 200 μg/mL concentration. *A. broughtonii* and *S. sachalinensis* cold water extract treatments at 200 μg/mL concentration showed a weak effect and the cell viabilities were 76.4 and 78.8%, respectively.

### 2.9. In Vivo Hepatoprotective Activity of C. japonica Hot Water Extract

Liver damage was assessed by biochemical studies with aspartate aminotransferase (AST) and alanine aminotransferase (ALT) and by the activity of free radical scavenging enzymes, such as superoxide dismutase (SOD), catalase (CAT) and glutathione peroxidase (GPX) in the CCl_4_ treated animals. Hepatoprotector Carsil (extract of *Silybum marianum*) was used as a reference drug.

Table 6 shows the changes in liver tissue weight of CCl_4_-treated rats with or without gavages of *C. japonica* extract. There was a significant increase (*p* < 0.05) in the relative liver weight gain in CCl_4_-induced rats when compared to the control group. No significant difference in the liver index was identified between the Carsil treatment group and the *C. japonica* extract treatment group (*p* > 0.05).

The effect of *C. japonica* extract on the CCl_4_-induced elevation of serum AST and ALT activities is shown in Table 7. Treatment with CCl_4_ significantly elevated the level of serum AST (9.7 mM/L) and ALT (11.6 mM/L) activities in rats compared to the control group (*p* < 0.05). Administration of *C. japonica* extract significantly decreased AST (5.7 mM/L) and ALT (4.5 mM/L) activities when compared to the CCl_4_-treated group.

The effect of *C. japonica* extracts on malondialdehyde (MDA), SOD, CAT, GPX levels is cited in Table 8. Results of the study clearly revealed an increase in the levels of MDA in CCl_4_-intoxicated rats comparing to the control group. Treatment with *C. japonica* extract significantly prevented this rise in levels. The GPX, SOD and CAT content has significantly increased in the extract treated group, whereas the CCl_4_-intoxicated group showed a significant decrease in levels comparing to the control group. The *C. japonica* treated group was as effective as the Carsil treated group.

The *C. japonica* extract was observed to exhibit a hepatoprotective effect as demonstrated by a significant decrease in AST and ALT concentrations. Moreover, the extract enhanced the activities of the antioxidant enzymes (SOD, CAT, GPX) and diminished the amount of lipid peroxide in comparison to the CCl_4_-induced hepatotoxicity in these animals. The hepatoprotective activity of the *C. japonica* extract was as strong as that of Carsil.

## 3. Discussion

*C. japonica*, *A. broughtonii*, and *S. sachalinensis* had high levels of protein, with their amino acid profile dominated by leucine, lysine, and phenylalanine. The amounts of isoleucine, threonine, and aromatic amino acids significantly confirmed the antioxidant activity of the tissue of these shellfish. The carbohydrate content was also low, as were the total lipid levels. In addition, *C. japonica*, *A. broughtonii*, and *S. sachalinensis* had high levels of calcium and iron. Overall, our results indicated that *C. japonica*, *A. broughtonii*, and *S. sachalinensis* had balanced nutritional qualities suitable for human consumption, and that their intake could contribute to a healthy and well-balanced diet. In addition, *A. broughtonii* and *S. sachalinensis*, can be used as dietary products due to their low fat content.

The antiradical ability was also recorded from cold and hot water extracts in all three species. The dependence of the radical scavenging activity of the extract on the molecular mass distribution of proteins and peptides was found. It was found that the amount of low molecular weight proteins and peptides (molecular weight less than 5 kDa) were significantly and positively correlated with the DPPH and ABTS radical scavenging activity. The *C. japonica* cold water extract was characterized by the highest amount of low molecular weight peptides, and its antiradical activity against DPPH and ATBS was the highest. However, the antiradical activity cannot be directly attributed to the number of low molecular weight peptides, since the decisive factor is the structure of the peptide and the sequence of its amino acids. The effect of the types and sequences of amino acids on their radical scavenging activity of bivalve tissue water proteins needs further studies.

In this paper, via the preliminary screening, the water extracts of bivalve tissues were evaluated for their hepatoprotective activity on t-BHP-induced acute liver injury in vitro. Among the different extracts the most promising results were found with the *C. japonica* tissue water extract. The dose-dependent *C. japonica* extract study revealed antihepatotoxic activity, especially at a dose of 100–200 mg/mL. The above-mentioned results revealed that *C. japonica* is valuable for traditional use for treating various liver diseases. In light of the distinct radical scavenging properties of *C. japonica* extract, the studies were further extended to in vivo conditions using CCl_4_-induced hepatotoxicity in rats. The hepatoprotective activity of the *C. japonica* extract was comparable to that of the known hepatoprotective drug Carsil. It is well known that antioxidant capacity is associated with hepatoprotective activity. Various in vivo and in vitro studies have shown that the hepatoprotective effect of natural compounds could be associated with the inhibition of oxidative stress by enhancing the antioxidant defense system [19,20]. The demonstration of both antioxidant and hepatoprotective activities by *C. japonica* may confirm this relationship.

In the tissues of *S. sachalinensis* and *A. broughtonii*, hepatoprotective activity was practically absent. However, these bivalve mollusks can be a valuable source of natural compounds, proteins, and minerals and can also be used as food ingredients. These species of shellfish can be valued not only for the texture and taste but also for their composition, which ensures a low calorie diet as they are low in fat.

## 4. Materials and Methods

### 4.1. Materials

Shellfish samples (*Corbicula japonica*, *Anadara broughtonii*, *Spisula sachalinensis*) were collected from the east coastal areas of Russia, Sea of Japan, Peter the Great Bay. *C. japonica* was collected with a dredge using a boat during the diurnal high tide period (N 43.321; E 131.771; 10 May 2019). *A. broughtonii* was collected with a dredge using a boat (N 43.160; E 131.480; 15 May 2019). *S. sachalinensis* by divers using scuba diving equipment (N 42.619; E 130.789; 12 May 2019). After the collection was collected, live *C. japonica* was washed with fresh running water; live *A. broughtonii* and *S. sachalinensis* were washed with running sea water. All clams were frozen and stored at −20 °C.

### 4.2. Chemical Analysis of Muscle Tissue

The moisture content of the clam samples was determined by the oven-drying method (100 °C for 18 h). In brief, a tissue sample (approximately 3 g) was dried in an oven at 105 °C for 6 h. Moisture content was expressed as percentages of the sample’s initial weight. Nitrogen content of samples was measured by total Kjeldahl Nitrogen method, (Kjeltec Auto 1030 Analyzer, Foss Tecator AB, Hoganas, Sweden). Nitrogen content was then multiplied by a factor to arrive at protein content. The average nitrogen (N) content of proteins found by the above method led to use of the calculation N × convert factor (6.25). Fat was extracted according to Bligh and Dyer [21], using a mixture of chloroform-methanol (1:2, *v*/*v*). The fat extract was weighed and the total lipid content was expressed as percentage of the dry muscle weight. For ash analysis, 2 g sample was placed in a crucible and pre-dried at 105 °C for 3 h. The sample in the crucible was then burned at 550 °C for 8 h. Protein, fat and ash contents were expressed as percentages of the sample’s dry weight.

Amino acids were quantified using the Beckman amino acid analyzer (model 6300, Beckman Coulter, Inc., Fullerton, CA USA). Major structural minerals: calcium, phosphorus, magnesium as well as the trace mineral, iron, were determined using inductively coupled plasma optical emission spectrometry (model P400, Perkin Elmer, Shelton, CT, USA).

Free amino acids were assessed using L-8900 Amino Acid Analyzer (Hitachi, Japan), with ion exchange column (ion exchange resin #2622). Before preparing the extracts for HPLC analyses, frozen tissues were lyophilized for 48 h, dry tissues were then powdered and weighed. Briefly, approx. 100 mg of dry tissue was suspended in 5 mL 0.2 M perchloric acid. The mixture was kept in an ultrasonic bath for 30 min and then centrifuged at 10,000× *g* for 20 min (Hitachi RX II). The supernatant was filtered through a 0.45 μm membrane (Whatman, PVDF). The peaks of samples were identified by comparing with elution times of standards (Sigma, St. Louis, MO, USA).

### 4.3. Aqueous Extraction

Distilled water was used as extraction solvent. Shellfish were opened with knife and edible portions, including hepatopancreas, muscle, gill, mantle and fluid were collected. Shellfish tissue was homogenized with cold distilled water in a 1:1 (*w*/*w*) ratio, in a high speed tissue homogenizer (Ika 25T basic, IKA Works Inc., Wilmington, NC, USA), at 4 °C. The obtained extracts were centrifuged at 8.000× *g* for 10 min (Hitachi CT 15RE) and the supernatant was collected and filtered (Whatman, 0.45 µm PVDF).

For hot extraction, a shell freshly caught and washed from sand and silt was poured with water in a ratio of 1:1 (*w*:*w*), heated to boiling, and boiled for 15 min. Then the solution was filtered and the water-soluble portion was collected.

### 4.4. Peptide Analysis by High-Performance Liquid Chromatography (HPLC)

The molecular mass distributions of extracts were performed in a modular Agilent Technologies liquid chromatograph (Agilent Technologies 1260 Infinity, USA, CA) with a UV detection at 280 nm. All samples were prepared twice and measured by duplicate. A TSK gel G 3000PWXL column, 7.8 mm I.D. × 30 cm (TOSOH Corporation, Tokyo, Japan) at a flow-rate of 0.3 mL/min and a temperature of 25 °C were used. The mobile phase consisted of 0.1 N NaCI 20 mM Tris-HCI buffer, pH 7.8. The approximate molecular weight (MW) was determined using standard protein samples (Sigma-Aldrich Co., USA, MO) as reference: bovine serum albumin (MW 66.3 kDa), egg albumin (MW 44.3 kDa), myoglobin (MW 18.0 kDa) cytochrome C (MW 12.4 kDa), aprotinin (MW 6.5 kDa), bacitracin (MW 1.4 kDa). All samples were filtered through 0.2 µm syringe filter (Whatman, PVDF) before injection. The MW of peptides was calculated by the elution time.

### 4.5. 2,2-Diphenyl-1-Picrylhydrazyl (DPPH) Radical Scavenging Activity

The scavenging effect on DPPH free radical was measured by the method of [22] with some modification. DPPH solution (200 μL, 0.1 mM in 96% ethanol) was mixed with 200 μL of extract. The mixture was shaken and left in the dark for 30 min. The absorbance of the resulting solution was measured at 517 nm using a Polarstar Omega microplate reader (BMG Labtech GmbH, Ottenberg, Germany).

Radical scavenging capacity was calculated as follows:

(1) $$\mathrm{DPPH}\;\mathrm{radical}\;\mathrm{scavenging}\;\mathrm{capacity}\;\left(\%\right)=\frac{\left(A_{\mathrm{control}}-{\;A}_{\mathrm{sample}}\right)}{A_{\mathrm{control}}}\times 100,$$

where “A_control_” is an absorbance of the reference solution (200μL of distilled water instead of the test sample), “A_sample_” is an absorbance of the test solution.

The DPPH radical scavenging activity was expressed in mg ascorbic acid/g water-soluble protein.

### 4.6. 2,2′-Azinobis(3-Ethylbenzothiazoline-6-Sulfonic Acid) (ABTS) Radical Scavenging Activity

ABTS radical scavenging activity was determined by method of Re et al. [23]. Trolox 2.5 mM (6-hydroxy-2,5,7,8-tetramethychroman-2-carboxylic acid) was used an antioxidant standard. The radical cation ABTS was produced by the reaction between the solution containing ABTS 7 mM with potassium persulfate 2.45 mM (final concentration) in a dark room for 12–16 h. During the measurement, an ABTS working solution was used by diluting the radical ABTS solution in phosphate buffer solution (pH = 7.4) until an absorbance at 734 nm of 0.70 ± 0.02 at room temperature of 25 °C, as determined using a Polarstar Omega microplate reader (BMG Labtech GmbH, Germany). A sample (15 µL) was mixed with 280 µL ABTS solution and the mixture was left in the dark for exactly 6 min at 25 °C. The absorbance at 734 nm was measured using a spectrophotometer. Decolorization of the assay was linear with the increasing concentrations of Trolox. ABTS radical scavenging activity was calculated as follows:

(2) $${\mathrm{ABTS}}^{+}\mathrm{radical}\;\mathrm{scavenging}\;\mathrm{capacity}\;\left(\%\right)=\frac{\left(A_{\mathrm{control}}-A_{\mathrm{sample}}\right)}{A_{\mathrm{control}}}\times 100$$

where “A_control_” is an absorbance of the reference solution (15 µL of distilled water instead of the test sample), “A_sample_” is an absorbance of the test solution. Trolox was used as the reference standard, and the results were expressed as µmol Trolox equivalent/g water-soluble protein.

### 4.7. Cell Viability Assay

For cell viability assay [24], HepG2 cells were dispensed into 96 well plates at the concentration of 1 × 10^4^ cells per well. After 24 h incubation, cells were cotreated with t-BHP (0.4 mM) and various concentrations of water extract of bivalve tissue (dissolved in 0.5% dimethylsulfoxide (DMSO)) for 3 h. The cells were added with 0.5 mg/mL MTT (3-(4,5-cimethylthiazol-2-yl)-2,5-diphenyl tetrazolium bromide) and incubated for another 4 h, and then the cell medium was replaced by 200 μL DMSO. Absorbance at 570 nm was determined with a Polarstar Omega microplate reader (BMG Labtech GmbH, Germany) and used for the measurement of the proportion of surviving cells.

### 4.8. Animals

Twenty-eight outbred albino rats weighing 280–300 g were used in this study. The animals were housed in the Pacific State Medical University animal center with sufficient air, a controlled temperature (22 ± 1 °C) and a 12-h light/dark cycle. The animals were supplied with food (standard rat chow) and water.

All animal experiments were carried out in accordance with the legislation of the Russian Federation and with the International document ETS no. 123 “European Convention for the Protection of Vertebrate Animals Used in Experiments and for Other Scientific Purposes” and with the approval of the Local Bioethics Committee.

### 4.9. Experimental Design and Biochemical Studies

Liver toxicity was induced in rats by per oral single administration of carbon tetrachloride and olive oil suspension (1:1, *v*/*v*) at a dose of 1.25 mL/kg [25].

Hepatoprotective Carsil (silymarin analog, Silybum marianum extract, Sopharma AO, Bulgaria) is a reference medication and was liquefied in saline and fed to the animals orally at a dose of 50 mg/kg. In terms of the amount of silymarin in this dosage form (22.5 mg per tablet of 500 mg), the animals received 2.25 mg/kg; this is comparable to the therapeutic dose for humans indicated in the drug leaflet.

All rats were randomly divided into four groups (I–IV) with seven rats each. The rats in group 1 serving as vehicle control received only olive oil.

Twenty-eight adult male rats haphazardly separated into four clusters with seven rats each.

Group 1: Normal rats fed with basal diet.
Group 2: Hepatic impaired rats were given water and CCl_4_-olive oil suspension.
Group 3: Hepatic impaired rats were treated with 50 mg/kg of the *C. japonica* extract and CCl_4_-olive oil suspension.
Group 4: Hepatic impaired rats were treated with 50 mg/kg of the Carsil (silymarin analog, *Silybum marianum* extract) and CCl_4_-olive oil suspension.

Water, *C. japonica* extract, Carsil and CCl_4_-olive oil suspension were given by gavages administration.

Carsil and *C. japonica* extract were administered 1 h before the administration of CCl_4_ every day for four days. On the second day after the last treatment, all rats were fasted overnight, weighed and recorded. Rats were then anesthetized with 5% chloral hydrate for collection of blood and liver tissues. Blood samples, from the abdominal aorta collected in a vacuum blood collection tube for biochemical analysis, were centrifuged for 5 min at 3500 r/min. The liver samples were quickly harvested and weighed, and the hepatic lobule was fixed in 4% paraformaldehyde for histopathological observation. The serum samples and other liver tissues were stored at −80 °C for study.

Serum samples were extracted from cardiac cell puncture by centrifugation at 3000 rpm for 15 min. Serum aminotransferase levels (ALT and AST) were measured using enzymatic kits from Olvex Diagnosticum (Russia). Livers were immediately excised from sacrificed rats and homogenized with ice-cold physiological saline. Then, the homogenate was centrifuged at 2500 rpm for 20 min at 4 °C. The supernatant was carried on for further analysis. MDA, SOD, CAT and GPX activities were measured using commercially available kits from Olvex Diagnosticum (Russia).

### 4.10. Statistical Analysis

The measurements were carried out three times and the data was analyzed using the software Statistica 7. The results were expressed as an average value with standard deviation. Values having a 95% confidence range (*p* < 0.05) were considered to be statistically significant ones.

## Figures and Tables

**Table 1:** Proximate composition of muscle tissue of bivalve mollusks (%).

Parameter	Mean Value ± SD		
	*C. japonica*	*S. sachalinensis*	*A. broughtonii*
Moisture *	89.0 ± 0.3	80.1 ± 0.2	80.2 ± 0.3
Dry matter	11.0 ± 0.3	19.9 ± 0.2	19.8 ± 0.3
Ash	10.0 ± 0.1	10.1 ± 0.1	8.6 ± 0.1
Protein	38.1 ± 0.3	61.1 ± 0.2	81.9 ± 0.2
Fat	33.2 ± 0.3	4.5 ± 0.1	3.8 ± 0.1
Carbohydrate	19.1 ± 0.3	24.2 ± 0.2	6.1 ± 0.3

* Moisture based on 100 g fresh weight; all other parameters based on 100 g dry weight.

**Table 2:** Amino acid prolife of bivalve mollusks tissue, g/100 g protein.

Amino Acid	*C. japonica*	*A. broughtonii*	*S. sachalinensis*	FAO/WHO
Histidine	3.61	1.83	1.94	1.5
Isoleucine	4.35	4.13	3.93	3.0
Leucine	6.90	7.05	6.51	5.9
Lysine	7.26	6.79	5.39	4.5
Methionine + Cysteine	1.95	3.64	1.14	2.2
Phenylalanine + Tyrosine	7.13	4.65	4.68	3.8
Threonine	5.28	4.24	3.92	2.3
Valine	5.02	4.76	4.12	3.9
Total EAAs	41.50	37.09	31.63	27.7

**Table 3:** Mineral content of the tissue of bivalve mollusks (µg/g wet weight).

Mineral	*C. japonica*	*A. broughtonii*	*S. sachalinensis*
Ca	534.4 ± 12.5	328.5 ± 21.5	171.5 ± 15.2
Mg	359.2 ± 9.1	280.3 ± 8.6	228.3 ± 12.3
Na	1692.5 ± 24.3	1256.3 ± 35.6	1456.8 ± 25.6
K	4203.5 ± 45.6	3970.7 ± 35.4	3561.4 ± 56.2
Fe	43.2 ± 6.3	65.2 ± 8.9	57.1 ± 2.5
Mn	2.6 ± 0.8	1.5 ± 0.4	0.6 ± 0.1
Zn	45.6 ± 2.5	12.4 ± 1.7	13.2 ± 2.1
Cu	2.7 ± 0.9	1.6 ± 0.5	1.6 ± 0.4

**Table 4:** The correlation coefficients between the amount of the four protein groups and the total radical scavenging activity of the water extracts of bivalve tissue.

Molecular Weight Group	Pearson’s Correlation Coefficient		*p*-Value		Determination Coefficient R^2^	
	DPPH	ABTS	DPPH	ABTS	DPPH	ABTS
Group I	0.1436	0.0559	0.0206	0.0031	0.7861	0.9163
Group II	−0.8591	−0.8166	0.0284	0.6669	0.7381	0.0474
Group III	0.9633	0.9570	0.0020	0.0027	0.9280	0.9158
Group IV	0.9144	0.9374	0.0107	0.0057	0.8362	0.8788

**Table 5:** The DPPH radical scavenging activity of the molecular fractions of the water extracts of bivalve tissue.

Bivalve Tissue	DPPH Radical Scavenging Activity µg Ascorbic Acid/g WS Protein			
	Group I	Group II	Group III	Group IV
*C. japonica*, cold extract	n/d *	5.9 ± 0.2	17.5 ± 0.5	10.8 ± 0.3
*C. japonica*, hot extract	n/d	3.8 ± 0.1	11.3 ± 0.3	6.2 ± 0.2
*A. broughtonii*, cold extract	n/d	1.1 ± 0.1	7.7 ± 0.2	3.2 ± 0.1
*A. broughtonii*, hot extract	n/d	n/d	2.5 ± 0.1	1.9 ± 0.1
*S. sachalinensis*, cold extract	n/d	1.2 ± 0.2	8.1 ± 0.2	4.1 ± 0.2
*S. sachalinensis*, hot extract	n/d	n/d	4.6 ± 0.2	2.6 ± 0.3

*—n/d—not detected.

**Table 6:** Relative liver weight of CCl_4_-treated rats with and without the gavage of *C. japonica* extract.

Group	Relative Liver Weight (g/100 g Body Weight)
Control	4.1 ± 0.2
CCl_4_	5.4 ± 0.3
*C. japonica* extract + CCl_4_	4.2 ± 0.1
Carsil + CCl_4_	4.6 ± 0.1

**Table 7:** Effect of *C. japonica* extract on CCl_4_ induced elevation in AST and ALT levels.

Group	ALT, mM/L	AST, mM/L
Control	2.7 ± 0.3	4.5 ± 0.1
CCl_4_	11.6 ± 1.4 *	9.7 ± 0.8 *
*C. japonica* extract + CCl_4_	4.5 ± 0.6 **	5.7 ± 0.5 **
Carsil + CCl_4_	3.6 ± 0.5 **	5.3 ± 0.6 **

Values are mean ± SD of 5–6 rats. * Significantly different from the control group, *p* < 0.05. ** Significantly different from the group treated with CCl_4_ only, *p* < 0.05.

**Table 8:** Effect of *C. japonica* extract on rat liver MDA, SOD, CAT and GPX in CCl_4_ induced hepatotoxicity in rats.

Group	MDA, nmol/mg Protein	SOD, U/mg Protein	CAT, U/mg Protein	GPX, U/mg Protein
Control	1.72 ± 0.13	125.6 ± 5.2	287.3 ± 7.9	0.84 ± 0.04
CCl_4_	5.12 ± 0.27 *	58.1 ± 3.3 *	195.1 ± 6.1 *	0.34 ± 0.01 *
*C. japonica* extract + CCl_4_	2.24 ± 0.17 **	118.3 ± 4.1 **	263.7 ± 5.5 **	0.68 ± 0.07 **
Carsil + CCl_4_	1.28 ± 0.19 **	109.9 ± 6.1 **	274.7 ± 7.4 **	0.75 ± 0.04 **

Values are mean ± SD of 5–6 rats. * Significantly different from the control group, *p* < 0.05. ** Significantly different from the group treated with CCl_4_ only, *p* < 0.05.

## Data Availability

Not applicable.

## Supplementary Materials

Figure S1: HPLC and molecular weight distribution of water-soluble proteins of *Anadara broughtonii* tissue (cold water extraction). Table S1. Molecular weight distribution of water-soluble proteins of Anadara broughtonii tissue (cold water extraction). Figure S2: HPLC and molecular weight distribution of water-soluble proteins of *Anadara broughtonii* tissue (hot water extraction). Table S2. Molecular weight distribution of water-soluble proteins of *Anadara broughtonii* tissue (hot water extraction). Figure S3: HPLC and molecular weight distribution of water-soluble proteins of *Spisula sachalinensis* tissue (cold water extraction). Table S3. Molecular weight distribution of water-soluble proteins of *Spisula sachalinensis* tissue (cold water extraction). Figure S4: HPLC and molecular weight distribution of water-soluble proteins of *Spisula sachalinensis* tissue (hot water extraction). Table S4. Molecular weight distribution of water-soluble proteins of *Spisula sachalinensis* tissue (hot water extraction). Figure S5: HPLC and molecular weight distribution of water-soluble proteins of *Corbicula japonica* tissue (cold water extraction). Table S5. Molecular weight distribution of water-soluble proteins of *Corbicula japonica* tissue (cold water extraction). Figure S6: HPLC and molecular weight distribution of water-soluble proteins of *Corbicula japonica* tissue (hot water extraction). Table S6. Molecular weight distribution of water-soluble proteins of *Corbicula japonica* tissue (hot water extraction).

## Sample Availability

Samples of the compounds are not available from the authors.
