# Supplementary material for: Proximate Composition, Antioxidant Properties, and Hepatoprotective Activity of Three Species of Shellfish of the Pacific Coast of Russia
Source: Molecules. 2021 Jun 3;26(11):3397. doi: 10.3390/molecules26113397 (PMC8199966; doi:10.3390/molecules26113397)
Supplement: Supplementary file 1 [file molecules-26-03397-s001.zip › molecules-1217061-supplementary.pdf]

Supporting Materials

Proximate Composition, Antioxidant Properties, and Hepatoprotective Activity of Three Species of Shellfish of the Pacific Coast of Russia

Ekaterina P. Karaulova *, Evgeny V. Yakush, Tatiana N. Slutskaya and Lidiya V. Shulgina

Russian Federal Research Institute of Fisheries and Oceanography, Pacific Branch (TINRO), 4, Shevchenko Alley, 690091 Vladivostok, Russia

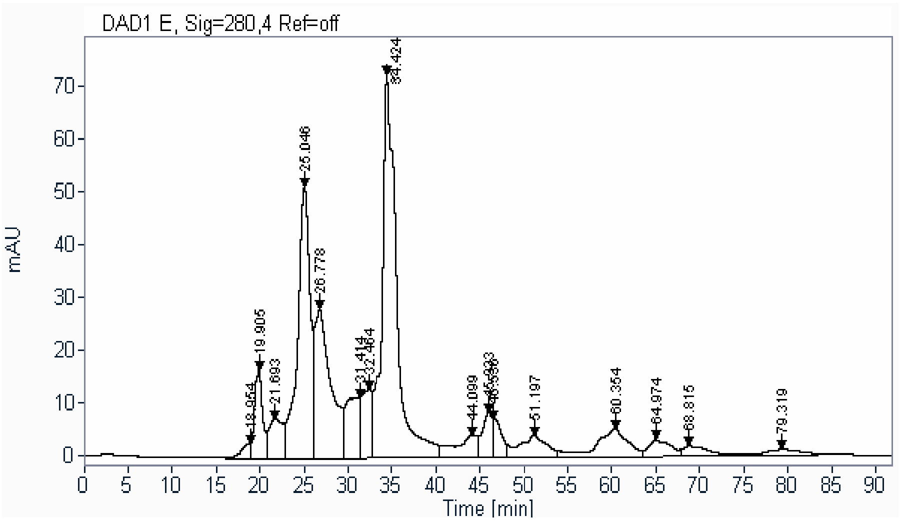

Figure S1. HPLC and molecular weight distribution of water-soluble proteins of *Anadara broughtonii* tissue (cold water extraction).

Table S1. Molecular weight distribution of water-soluble proteins of Anadara broughtonii tissue (cold water extraction).

№	RT [min]	Area%	RT ml	MW, Da
1	18,95	0,82	5,69	
2	19,91	3,97	5,97	>30000
3	21,69	3,19	6,51	
4	25,05	18,75	7,52	>10000
5	26,78	13,45	8,03	
6	31,41	4,71	9,42	7000
7	32,46	3,86	9,74	6800
8	34,42	31,08	10,33	6400
9	44,1	2,8	13,23	3900
10	45,93	2,51	13,78	3500
11	46,54	1,7	13,96	3300
12	51,2	3,32	15,36	2100
13	60,35	4,86	18,11	
14	64,97	2,1	19,49	<1000
15	68,82	1,58	20,65	
16	79,32	1,31	23,80	

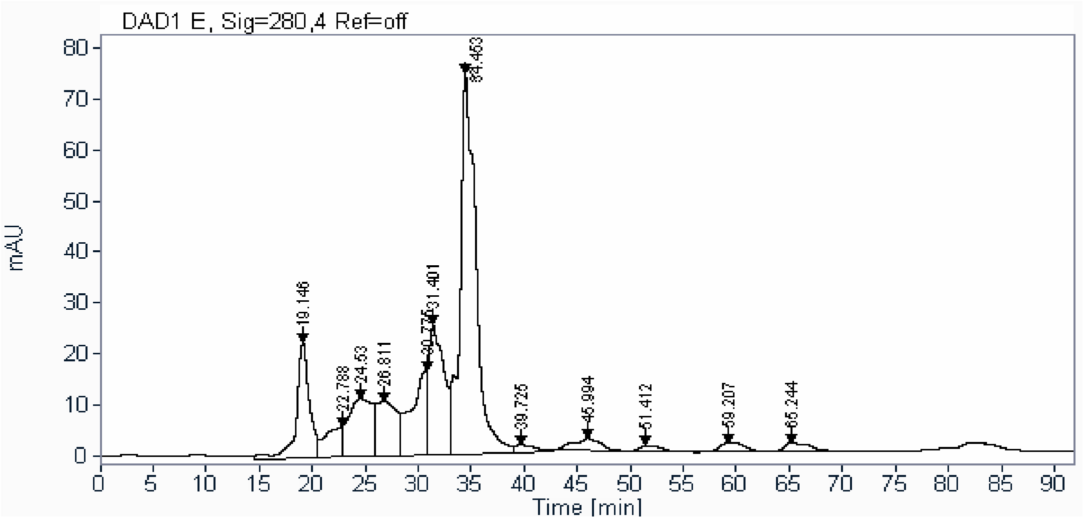

Figure S2. HPLC and molecular weight distribution of water-soluble proteins of *Anadara broughtonii* tissue (hot water extraction).

Table S2. Molecular weight distribution of water-soluble proteins of *Anadara broughtonii* tissue (hot water extraction).

№	RT [min]	Area%	RT ml	MW, Da
1	19,15	10,23	5,75	
2	22,79	3,36	6,84	>30000
3	24,53	9,44	7,36	
4	26,81	7,38	8,04	>10000
5	30,77	8,73	9,23	7300
6	31,4	13,71	9,42	7100
7	34,45	40,43	10,34	6400
8	39,72	1,01	11,92	5000
9	45,99	2,12	13,80	3500
10	51,41	0,8	15,42	2100
11	59,21	1,42	17,76	<1000
12	65,24	1,36	19,57	

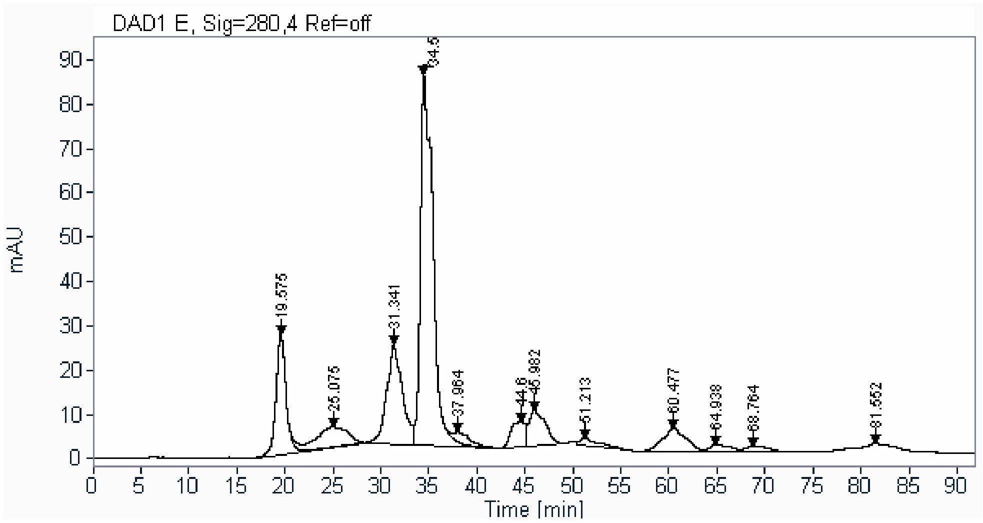

Figure S3. HPLC and molecular weight distribution of water-soluble proteins of *Spisula sachalinensis* tissue (cold water extraction).

Table S3. Molecular weight distribution of water-soluble proteins of *Spisula sachalinensis* tissue (cold water extraction).

№	RT [min]	Area%	RT ml	MW, Da
1	19,58	11,36	5,87	>30000
2	25,08	6,38	7,52	>10000
3	31,34	14,49	9,40	7500
4	34,5	44,19	10,35	6400
5	37,97	1,76	11,39	5500
6	44,6	3,26	13,38	3800
7	45,98	5,37	13,79	3500
8	51,21	1,42	15,36	2200
9	60,48	5,03	18,14	<1000
10	64,94	1,37	19,48	
11	68,76	1,29	20,63	
12	81,55	4,08	24,47	

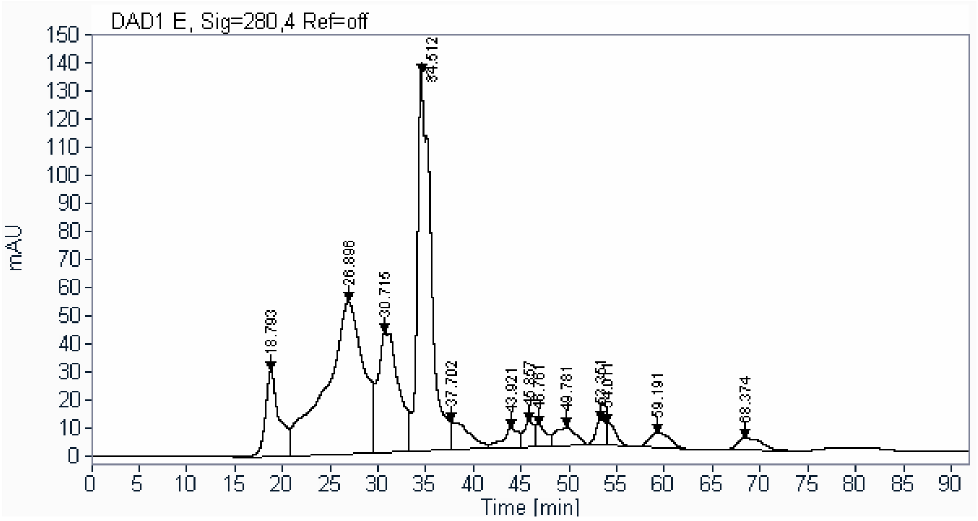

Figure S4. HPLC and molecular weight distribution of water-soluble proteins of *Spisula sachalinensis* tissue (hot water extraction).

Table S4. Molecular weight distribution of water-soluble proteins of *Spisula sachalinensis* tissue (hot water extraction).

№	RT [min]	Area%	RT ml	MW, Da
1	18,79	6,94	5,64	>30000
2	26,9	32,74	8,07	>10000
3	30,71	15,82	9,21	7300
4	34,51	31,9	10,35	6400
5	37,7	4,92	11,31	5600
6	43,92	0,56	13,18	4000
7	45,86	0,78	13,76	3500
8	46,71	0,74	14,01	3300
9	49,78	0,65	14,93	2500
10	53,35	0,8	16,01	1600
11	54,01	0,87	16,20	1500
12	59,19	1,8	17,76	<1000
	68,37	1,63	20,51	

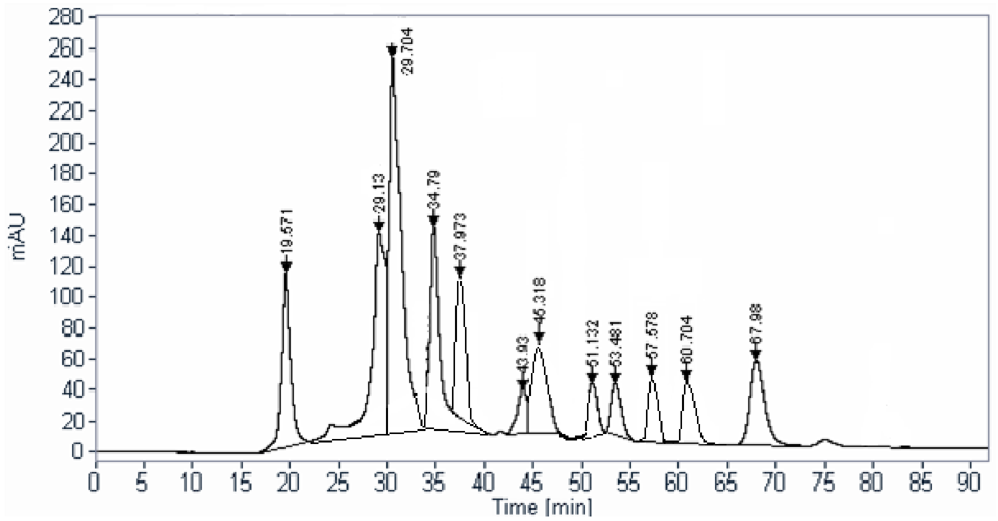

Figure S5. HPLC and molecular weight distribution of water-soluble proteins of *Corbicula japonica* tissue (cold water extraction).

Table S5. Molecular weight distribution of water-soluble proteins of *Corbicula japonica* tissue (cold water extraction).

RT ml	RT [min]	Area,%	MW
5,868	19,56	7,9	>30000
8,739	29,13	9,76	>10000
8,9112	29,704	18,92	
10,437	34,79	14,71	6300
11,391	37,97	12,36	5500
13,179	43,93	1,94	4000
13,596	45,32	9,62	3700
15,339	51,13	3,53	2200
16,044	53,48	3,49	1600
17,274	57,58	3,79	<1000
18,213	60,71	3,91	
20,394	67,98	9,97	

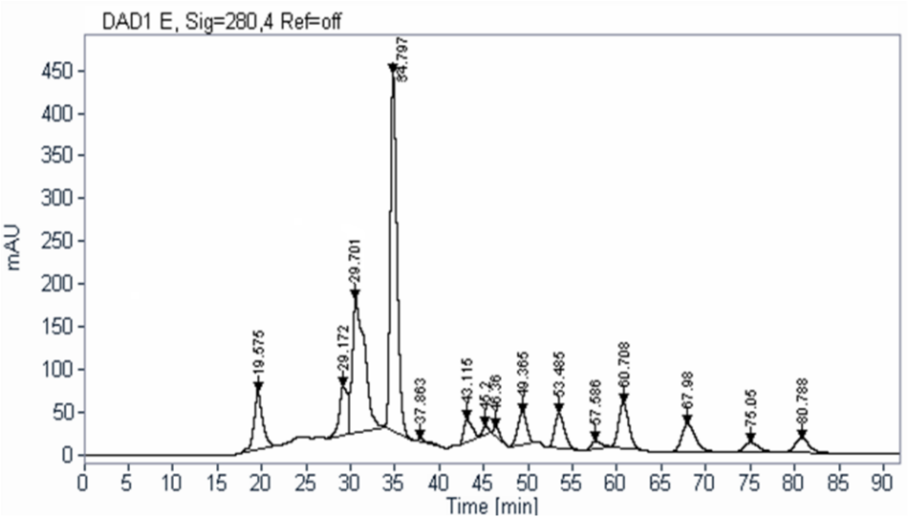

Figure S6. HPLC and molecular weight distribution of water-soluble proteins of *Corbicula japonica* tissue (hot water extraction).

Table S6. Molecular weight distribution of water-soluble proteins of *Corbicula japonica* tissue (hot water extraction).

RT min	Area, %	RT ml	MW
19,56	6,81	5,87	>30000
29,17	5,24	8,75	>10000
29,701	23,18	8,91	
34,8	34,98	10,44	6300
37,86	0,93	11,36	5500
43,11	2,31	12,93	4200
45,2	0,63	13,56	3700
46,36	0,64	13,91	3400
49,36	3,8	14,81	2600
53,49	4,2	16,05	1600
57,59	1,01	17,28	<1000
60,71	6,32	18,21	
67,98	5,1	20,39	
75,05	2,04	22,52	
80,79	2,71	24,24	
